# Genome-wide association analysis reveals potential genetic correlation and causality between circulating inflammatory proteins and amyotrophic lateral sclerosis

**DOI:** 10.18632/aging.205878

**Published:** 2024-05-30

**Authors:** Jing Shen, Xiaochu Gu, Chenxu Xiao, Hanfei Yan, Yu Feng, Xiaowei Li

**Affiliations:** 1 The Affiliated Jiangsu Shengze Hospital of Nanjing Medical University, Suzhou 251221, China; 2 Medical Laboratory, Suzhou Psychiatric Hospital, The Affiliated Guangji Hospital of Soochow University, Suzhou 215137, China; 3 The University of New South Wales, Sydney 2052, Australia; 4 The University of Melbourne, Melbourne 3010, Australia

**Keywords:** amyotrophic lateral sclerosis (ALS), circulating inflammatory proteins, genome-wide association study (GWAS), Mendelian randomization, neuroinflammation

## Abstract

Background: Amyotrophic Lateral Sclerosis (ALS), a fatal neurodegenerative disease, continues to elude complete comprehension of its pathological underpinnings. Recent focus on inflammation in ALS pathogenesis prompts this investigation into the genetic correlation and potential causal relationships between circulating inflammatory proteins and ALS.

Methods: Genome-wide association study (GWAS) data encompassing 91 circulating inflammatory protein measures from 14,824 individuals of European ancestry, alongside records from 27,205 ALS cases and 110,881 controls, were employed. Assessment of genetic correlation and overlap utilized LD score regression (LDSC), high-definition likelihood (HDL), and genetic analysis integrating pleiotropy and annotation (GPA) methodologies. Identification of shared genetic loci involved pleiotropy analysis, functional mapping and annotation (FUMA), and co-localization analysis. Finally, Mendelian randomization was applied to probe causal relationships between inflammatory proteins and ALS.

Results: Our investigation revealed significant genetic correlation and overlap between ALS and various inflammatory proteins, including C-C motif chemokine 28, Interleukin-18, C-X-C motif chemokine 1, and Leukemia inhibitory factor receptor (LIFR). Pleiotropy analysis uncovered shared variations at specific genetic loci, some of which bore potential harm. Mendelian randomization analysis suggested that alterations in specific inflammatory protein levels, notably LIFR, could impact ALS risk.

Conclusions: Our findings uncover a genetic correlation between certain circulating inflammatory proteins and ALS, suggesting their possible causal involvement in ALS pathogenesis. Moreover, the identification of LIFR as a crucial protein may yield new insights into ALS pathomechanisms and offer a promising avenue for therapeutic interventions. These discoveries provide novel perspectives for advancing the comprehension of ALS pathophysiology and exploring potential therapeutic avenues.

## INTRODUCTION

Amyotrophic Lateral Sclerosis (ALS), a progressive neurodegenerative disorder, predominantly impacts motor neurons, leading to muscle wasting and diminished strength [1–3]. In recent years, increasing attention has been directed towards the role of systemic inflammation in the pathogenesis of neurodegenerative disorders, with ALS being particularly devastating. While the precise etiology of ALS remains elusive, emerging evidence implicates genetic factors in its pathogenesis. ALS patients harboring genetic variations may manifest distinct clinical features and inheritance patterns, yet the correlation between genotype and phenotype remains somewhat ambiguous. Research underscores the importance of investigating pathogenic genes in ALS for diagnosis and the identification of potential drug targets [4, 5]. Recently, mounting evidence suggests a potential link between inflammatory processes and the progression of ALS, especially regarding the putative role of circulating inflammatory proteins. Circulating inflammatory proteins, serving as biomarkers of systemic inflammation (including cytokines, chemokines, and acute-phase reactants), not only undergo changes in ALS patients but also correlate with disease severity and progression. ALS, as a multifactorial, multisystem neuroinflammatory disorder, leads to compromised muscle function and eventual mortality. The onset and progression of ALS coincide with alterations in inflammatory proteins, while neuroinflammation further hastens disease progression and exacerbates its severity; however, peripheral inflammatory processes remain insufficiently characterized [6–8].

Genome-wide association studies (GWAS) represent a potent tool for elucidating the genetic underpinnings of complex diseases. By analyzing genetic data from large cohorts of cases and controls, GWAS have the capacity to identify genetic variants associated with disease risk. In ALS research, GWAS have successfully pinpointed multiple genetic loci linked to disease risk [9–11]. However, existing research on the genetic correlation and potential causality between circulating inflammatory proteins and ALS is still insufficient.

Inflammatory proteins, including cytokines and chemokines, serve as crucial components of the immune system, playing pivotal roles in modulating inflammatory responses and immune reactions. In ALS, aberrant expression of inflammatory proteins may impact the survival and function of neurons, thereby fostering disease progression [12–14]. The significance of these circulating proteins lies not only in their potential as diagnostic or prognostic markers but also in their ability to unveil novel therapeutic targets. For instance, alterations in specific inflammatory protein levels in ALS may be associated with crucial pathological processes such as glial cell activation, central nervous system immune cell infiltration, and neuronal death. This suggests that modulating the levels or activity of these proteins could ameliorate neuroinflammation and potentially slow disease progression. Therefore, a comprehensive understanding of the relationship between circulating inflammatory proteins and ALS is crucial for elucidating the disease's pathological mechanisms and developing novel therapeutic strategies.

This study leverages large-scale GWAS data in conjunction with sophisticated statistical and genetic analysis techniques, with the objective of investigating the genetic correlation between circulating inflammatory proteins and ALS, as well as evaluating their potential causal relationship. Through these analyses, our aim is to offer fresh insights into the genetic underpinnings and pathological mechanisms of ALS, thereby laying a scientific groundwork for the development of future therapeutic interventions.

## MATERIALS AND METHODS

### The research process is illustrated in the flowchart figure (Figure 1)

**Figure 1:**
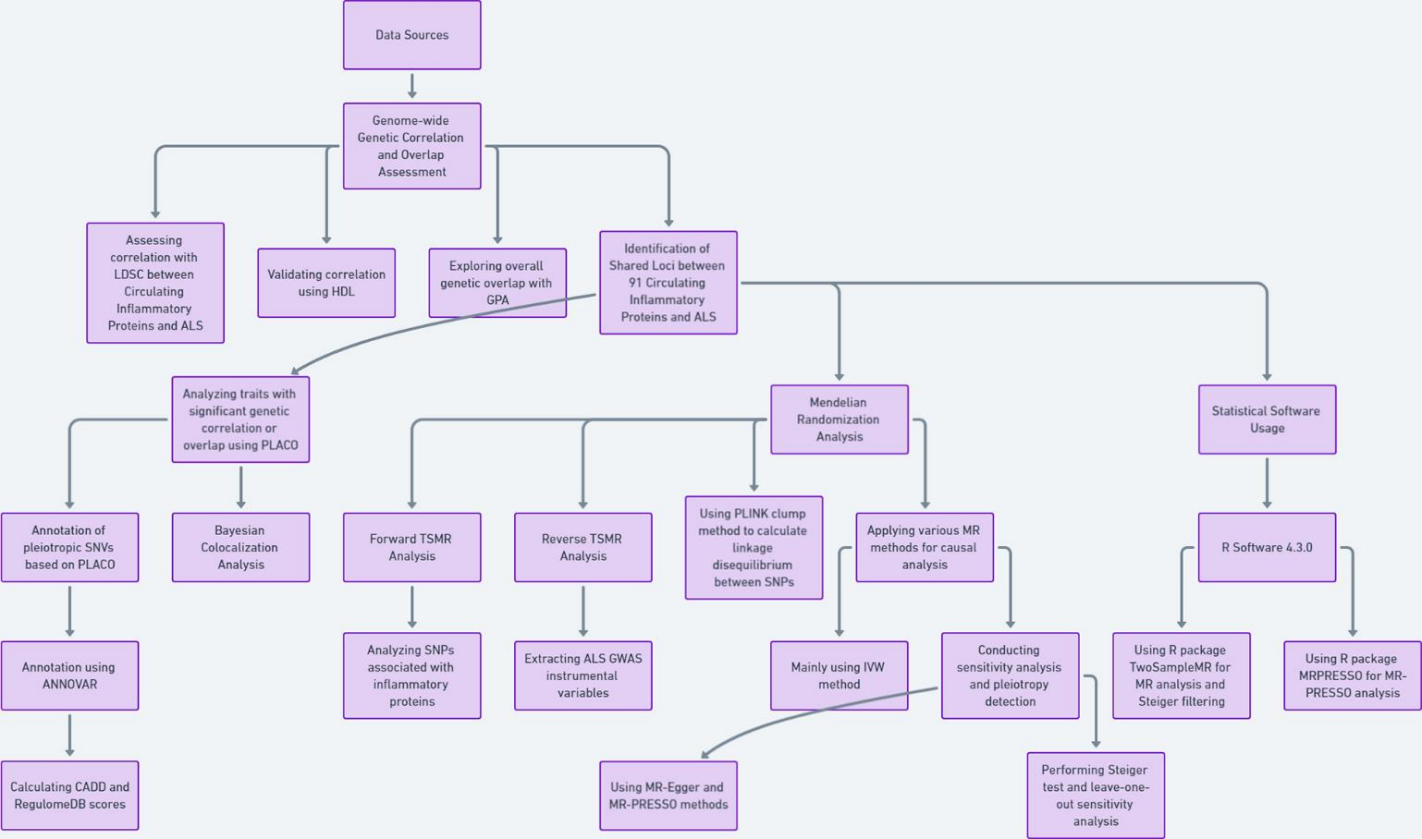
Flowchart figure.

### Data sources

The GWAS data for 91 circulating inflammatory proteins were derived from a total of 14,824 participants of European descent across 11 cohorts, as measured using the Olink Target Inflammation panel, reported by Zhao et al. [15]. The ALS data came from a meta-analysis of a genome-wide association study (GWAS) “GCST90027164” (https://www.ebi.ac.uk/gwas/studies/GCST90027164) by van Rheenen et al., which included 27,205 European ALS cases and 110,881 European controls [9] (Supplementary Table 1).

### Genome-wide genetic correlation and overlap

Initially, we employed linkage disequilibrium score regression (LDSC) to assess the genome-wide genetic correlation between the 91 circulating inflammatory proteins and ALS for 91 trait pairs [16]. For the LDSC analysis, we used LD scores based on European ancestry from the 1000 Genomes Project [17]. In our LDSC analysis, we did not restrict the intercept. Although sample overlap can affect the intercept, it does not influence the slope. Therefore, genetic correlation is not impacted in the case of sample overlap. This approach not only considers residual confounding factors but also indicates potential sample overlap between two GWAS studies. Subsequently, we utilized high-definition likelihood (HDL) to re-validate the genome-wide genetic correlation for these 91 trait pairs. HDL offers greater precision in estimating genetic correlations compared to LDSC [18].

Lastly, we used Genetic analysis incorporating Pleiotropy and Annotation (GPA) to explore the overall genetic overlap between traits. GPA integrates multiple GWAS datasets and functional annotations to identify association signals. GPA not only detects many subtle signals that traditional single-phenotype analyses might miss but also reveals relationships between their genetic structures [19]. For all the aforementioned analyses, the Bonferroni-corrected significance threshold was set at P < 0.05.

### Shared loci between 91 circulating inflammatory proteins and amyotrophic lateral sclerosis

For trait pairs showing significant genetic correlation or overlap, we employed pleiotropic analysis under composite null hypothesis (PLACO) to identify potential pleiotropic single-nucleotide variations (SNVs). PLACO specifically detects pleiotropic gene loci between two traits by considering the potential composite null hypothesis (i.e., a variant is unrelated to any trait or only related to one of the traits) [20]. SNVs with P. PLACO < 5 × 10^-8 were considered significant pleiotropic variations.

For pleiotropic SNVs identified by PLACO, we utilized functional mapping and annotation of genetic associations (FUMA) for further analysis to recognize independent variations [21]. FUMA delineates genomic risk loci and annotates variations' functions using LD information from the European population reference panel of the 1000 Genomes Project Phase 3. We set the Maximum P-value of lead SNV to less than 5 × 10^-8 and the Maximum P-value cutoff to less than 0.05. Independent SNVs within 1 Mb with r2 less than 0.6 and lead SNVs with r2 less than 0.1 were characterized. If the physical distance between lead SNVs was less than 250 kb, genomic risk loci were defined by merging genomic regions [22].

For pleiotropic SNVs identified based on PLACO, we annotated them using ANNOVAR [23] and calculated combined annotation-dependent depletion (CADD) and Regulome DB scores. SNVs with a CADD score greater than 12.37 were considered potentially harmful variations [24]. Regulome DB scores SNPs, with lower scores indicating stronger regulatory potential [25].

For these potential pleiotropic loci, we conducted Bayesian colocalization analysis to test hypotheses where H3 represents both traits being associated but with different causal variants, and H4 represents both traits being associated and sharing a causal variant. We used the default settings of the coloc.abf function (p1 = p2 = 1 × 10^-4, p12 = 1 × 10^-5), where p1 and p2 represent the prior probabilities of an SNP being associated with trait 1 and trait 2 respectively, and p12 represents the prior probability of an SNP being associated with both traits. A posterior probability (PP.H4) greater than 0.7 was used as the criterion for including a locus as colocalized [26, 27].

### Mendelian randomization

We employed two-sample Mendelian randomization (TSMR) analysis to investigate the causal relationship between circulating inflammatory proteins and ALS. In the MR analysis, inflammatory proteins serve as the exposure of interest, ALS as the outcome, and SNPs as instrumental variables (IVs). TSMR method is based on the following assumptions: (I) the instrumental variables are strongly associated with inflammatory proteins; (II) the instrumental variables only affect ALS risk through their association with inflammatory proteins; (III) the instrumental variables are independent of confounding factors [28, 29].

For the positive TSMR analysis, we selected SNPs associated with inflammatory proteins at a genome-wide significance level (P < 5×10^-8). For the reverse TSMR, SNPs from ALS GWAS were extracted, again using SNPs with P < 5×10^-8. Additionally, the PLINK clumping method was utilized to calculate linkage disequilibrium (LD) between each exposure SNP based on the 1000 Genomes European panel, with an r2 threshold of < 0.01 (clumping distance = 5000kb). The F-statistic was calculated using F = beta 2 /se 2. F-statistics > 10 indicate the robustness of IVs.

We employed several MR methods, including MR-Egger, weighted median, inverse variance-weighted (IVW), Wald ratio, simple mode, and weighted mode. IVW was selected as the primary analysis method, with Wald ratio used when SNPs < 2, and P-IVW values < 0.05 considered statistically significant [30, 31]. Cochran's Q statistic was used to assess heterogeneity among individual SNPs. If no significant heterogeneity was observed (P < 0.05), a fixed-effects model was employed [32]; otherwise, caution was exercised in interpreting causal significance. Sensitivity analyses were conducted to validate the robustness of our results. Furthermore, MR-Egger and MR-PRESSO methods were employed to assess for pleiotropy. The intercept obtained from MR-Egger regression was used to measure directional pleiotropy, while MR-PRESSO enhanced detection of pleiotropy [33]. Steiger tests were conducted to determine causality direction. Leave-one-out sensitivity analyses were performed to assess whether individual SNPs significantly influenced MR results.

### Statistical software

All statistical analyses were conducted in R software version 4.3.0 (https://www.r-project.org/). The “TwoSampleMR” package was used for MR analysis and Steiger filtering [34–36]. MR-PRESSO was conducted using the “MRPRESSO” package in R.

All analyses in this study were conducted after excluding SNVs in the MHC region (chromosome 6: 25-35 MB).

### Availability of data and materials

The GWAS data for 91 circulating inflammatory proteins were derived from a total of 14,824 participants of European descent across 11 cohorts, as measured using the Olink Target Inflammation panel, reported by Zhao et al. The Amyotrophic Lateral Sclerosis (ALS) data came from a meta-analysis of a genome-wide association study (GWAS) “GCST90027164” (https://www.ebi.ac.uk/gwas/studies/GCST90027164) by van Rheenen et al., which included 27,205 European ALS cases and 110,881 European controls.

## RESULTS

### Genetic correlation and overlap between 91 circulating inflammatory proteins and ALS

Among 91 paired traits, we observed significant genome-wide genetic correlations for three trait pairs identified by LDSC. Additionally, HDL identified significant genome-wide genetic correlations for eight trait pairs. Unfortunately, there were no trait pairs for which HDL and LDSC results were completely consistent (Supplementary Table 2). Traits such as C-C motif chemokine 28 levels (CCL28) and Interleukin-18 levels (IL18) showed positive genetic correlations in LDSC analyses (LDSC.rg = 0.222; 0.313), while CD40L receptor levels exhibited a negative genetic correlation (LDSC.rg = -0.429). We also noted that two of the three trait pairs identified by LDSC (CCL28 and IL18) showed significant genetic overlap in GPA analysis (P_GPA = 2.12E-8; 1.03E-07). Of the eight trait pairs identified by HDL, two, such as C-X-C motif chemokine 1 levels (CXCL1) and Leukemia inhibitory factor receptor levels (LIFR), also demonstrated significant genetic overlap in GPA analysis (P_GPA = 2.91E-2; 6.34E-32). The final joint set of these four traits will be used for subsequent analysis.

### Shared loci between four circulating inflammatory proteins and ALS

**Figure 2:**
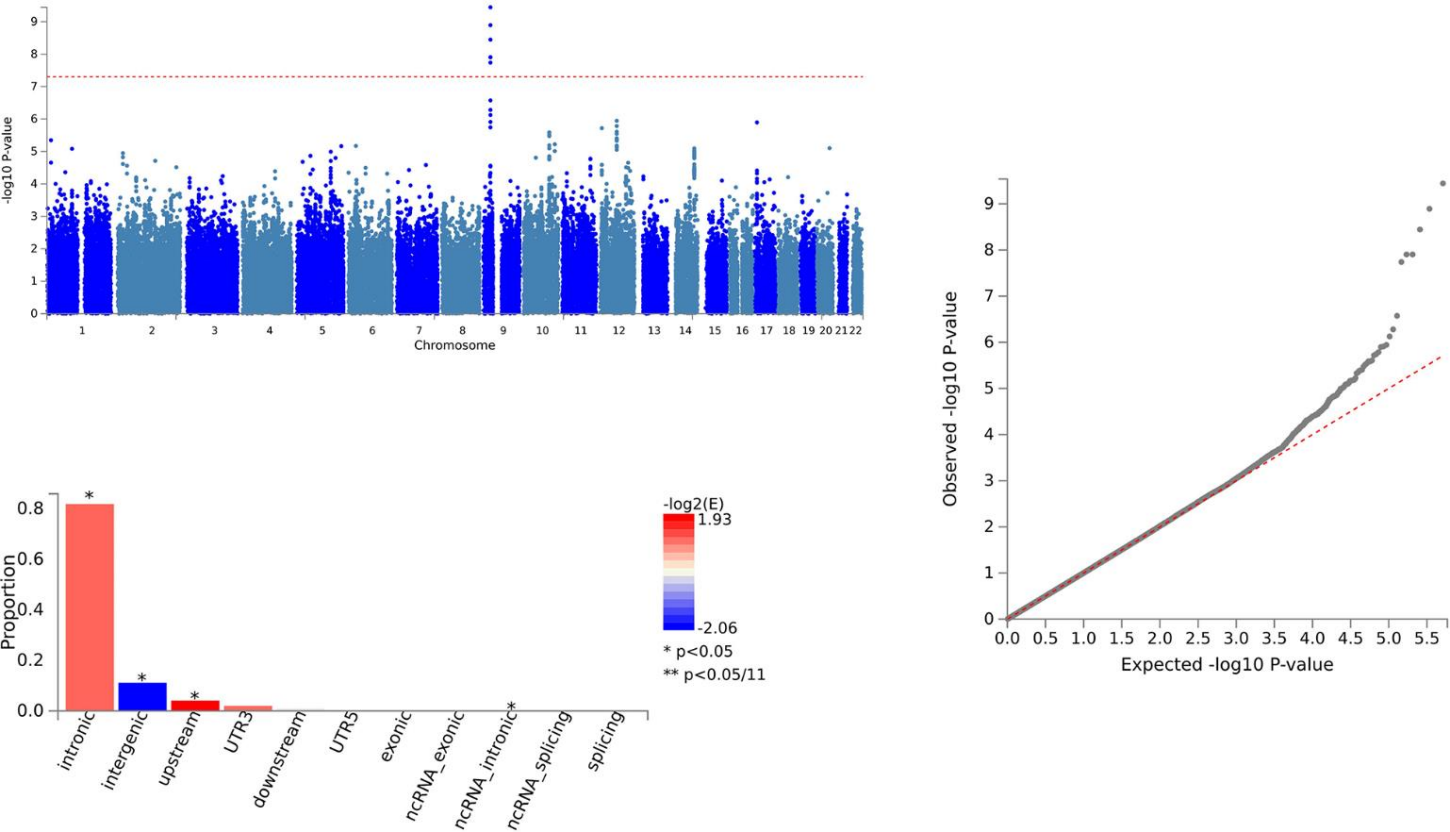
Shared sites between C-C motif chemokine 28 levels and ALS.

In the case of CCL28 and ALS, we identified 79 SNVs using PLACO and further delineated 64 independent genomic risk loci through FUMA, all located in chromosome region 9p21.2. Two shared SNVs were identified as potentially harmful variations: rs10967965 (CADD=17.2) and rs700795 (CADD=16.7) (Supplementary Table 3 and Figure 2).

**Figure 3:**
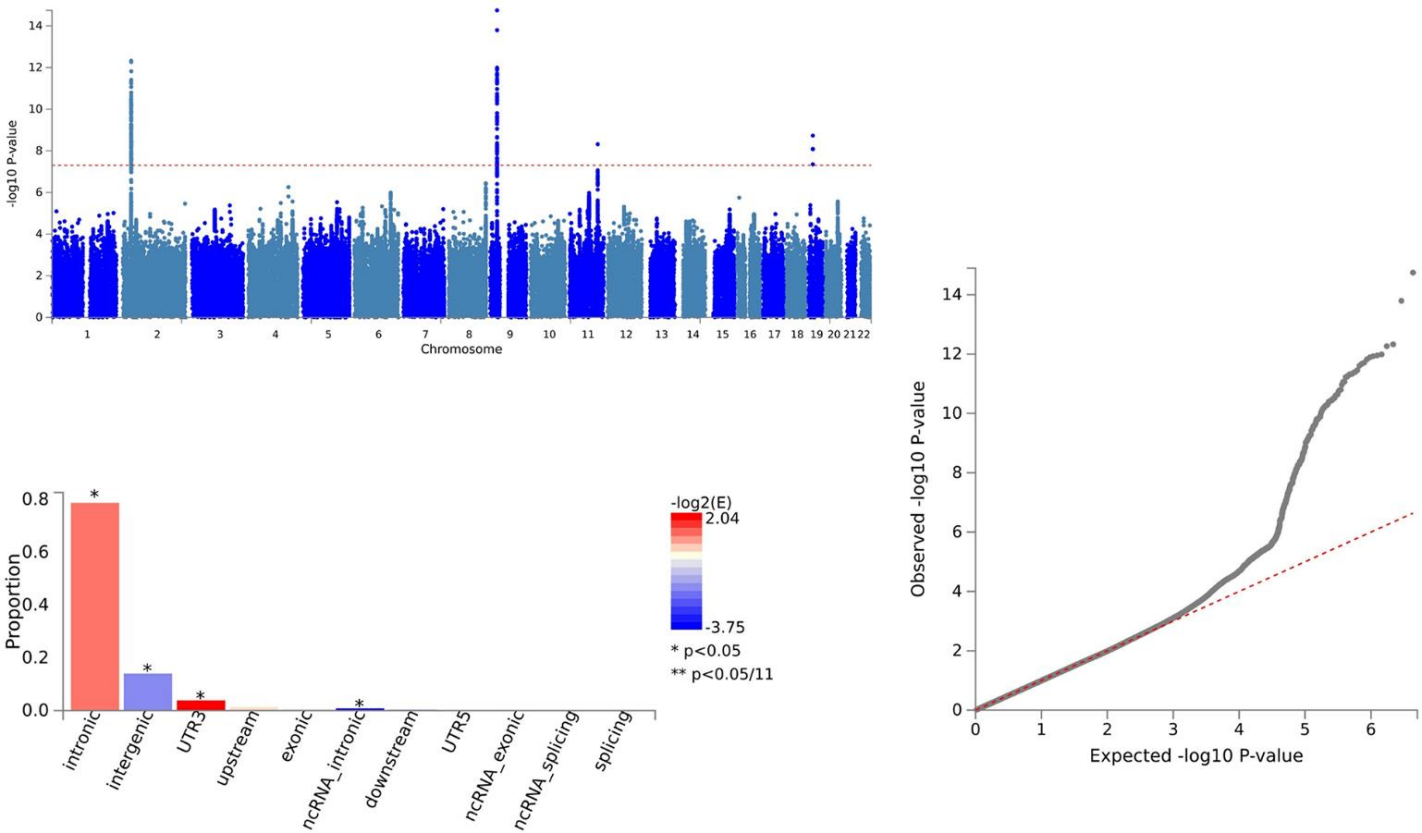
Shared sites between Interleukin-18 levels and ALS.

For IL18 and ALS, 157 SNVs were identified with PLACO, and FUMA confirmed 157 independent genomic risk loci across four chromosome regions: 2p22.3, 9p21.2, 11q23.1, and 19p13.11. Six shared SNVs were deemed potentially harmful: rs762019 (CADD=17.43), rs2366894 (CADD=17.61), rs868856 (CADD=16.3), rs10967965 (CADD=17.2), rs700795 (CADD=16.7), and rs77203424 (CADD=12.44) (Supplementary Table 4 and Figure 3).

**Figure 4:**
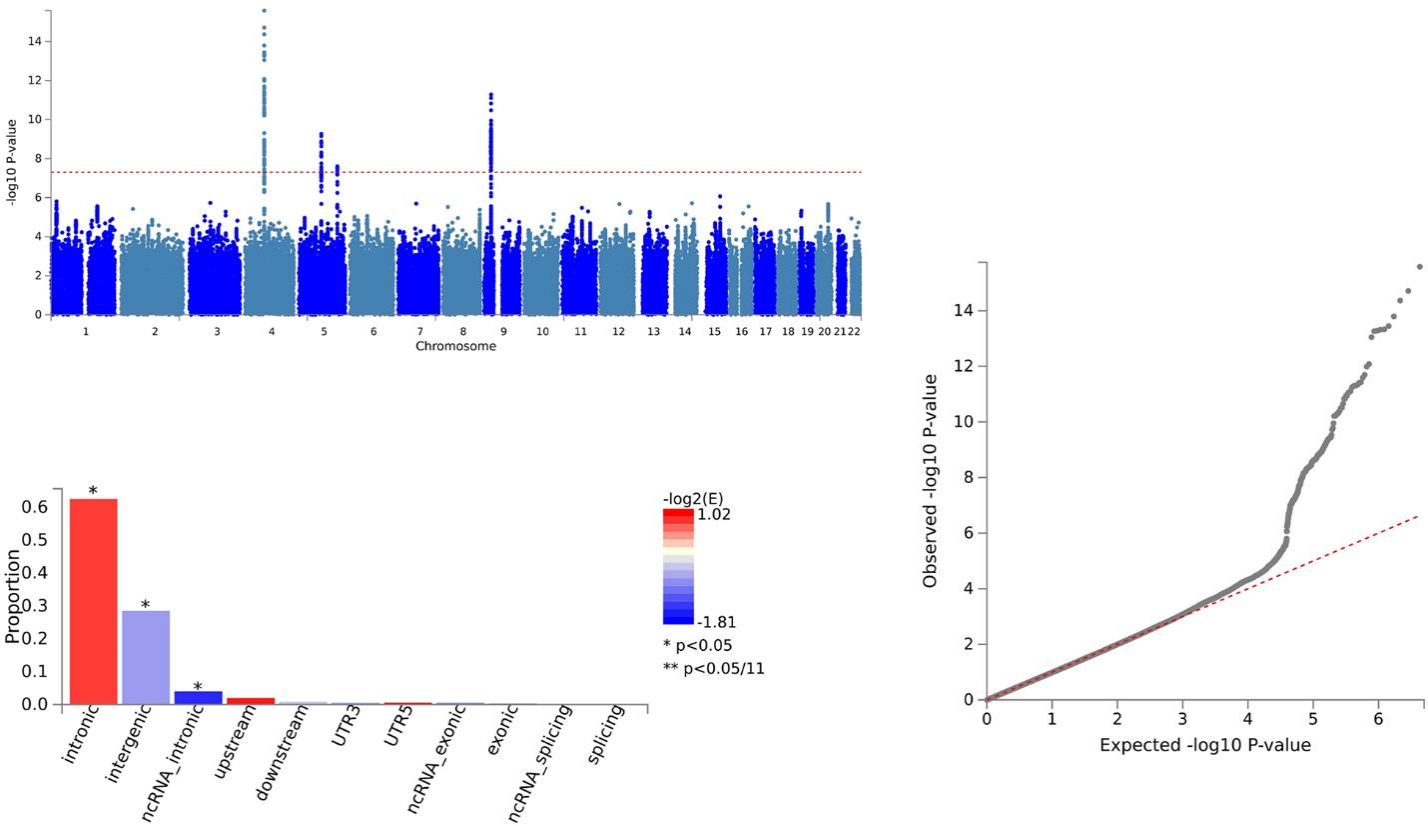
Shared sites between C-X-C motif chemokine 1 levels and ALS.

In the analysis of CXCL1 and ALS, 164 SNVs were identified via PLACO, and FUMA pinpointed 164 independent genomic risk loci in three chromosome regions: 4q13.3, 5q14.3, and 9p21.2. Five shared SNVs were considered potentially harmful: rs61104616 (CADD=17.99), rs4879515 (CADD=15.78), rs868856 (CADD=16.3), rs10967965 (CADD=17.2), and rs700795 (CADD=16.7) (Supplementary Table 5 and Figure 4).

**Figure 5:**
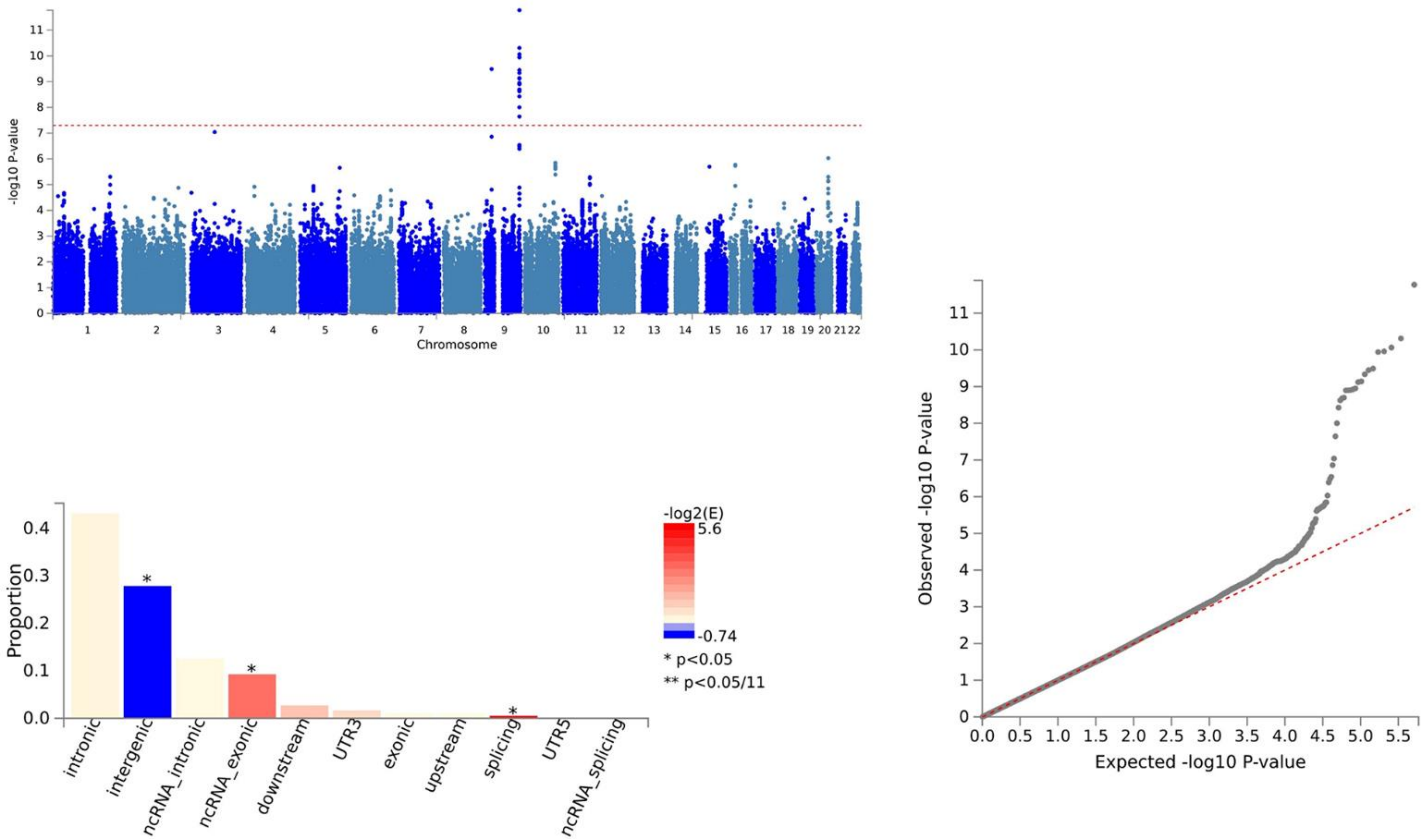
Shared sites between leukemia inhibitory factor receptor levels and ALS.

For LIFR and ALS, PLACO identified 22 SNVs, and FUMA established 21 independent genomic risk loci in two chromosome regions: 9p21.2 and 9q34.2. One shared SNV, rs10967965 (CADD=17.2), was identified as potentially harmful (Supplementary Table 6 and Figure 5).

Additionally, a common pleiotropic SNV (rs10967965) was identified across these trait pairs, with the nearest gene being MOB3B in region 9p21.2, indicating broad pleiotropy of this locus.

Through colocalization analysis, we identified one potential pleiotropic locus (5q14.3) with a PP.H4 greater than 0.7 (Supplementary Table 5). This finding indicates that the 5q14.3 locus plays a significant role in the shared pathogenesis of traits (CXCL1).

For CCL28 and ALS, pleiotropic loci were annotated using ANNOVAR. We found that 7 SNVs were intergenic variations, and 54 SNVs were intronic variations. There were no exonic variations identified (Supplementary Table 3 and Figure 2).

In the case of IL18 and ALS, pleiotropic loci were annotated using ANNOVAR. We identified 23 SNVs as intergenic variations, 122 SNVs as intronic variations, and 2 SNVs as exonic variations (Supplementary Table 4 and Figure 3).

For CXCL1 and ALS, pleiotropic loci were annotated using ANNOVAR. We found 60 SNVs as intergenic variations, 91 SNVs as intronic variations, and 1 SNV as an exonic variation (Supplementary Table 5 and Figure 4).

In the analysis of LIFR and ALS, pleiotropic loci were annotated using ANNOVAR. We identified 11 SNVs as intergenic variations, 1 SNV as an intronic variation, and 1 SNV as an exonic variation (Supplementary Table 6 and Figure 5).

### Forward Mendelian randomization results

**Figure 6:**
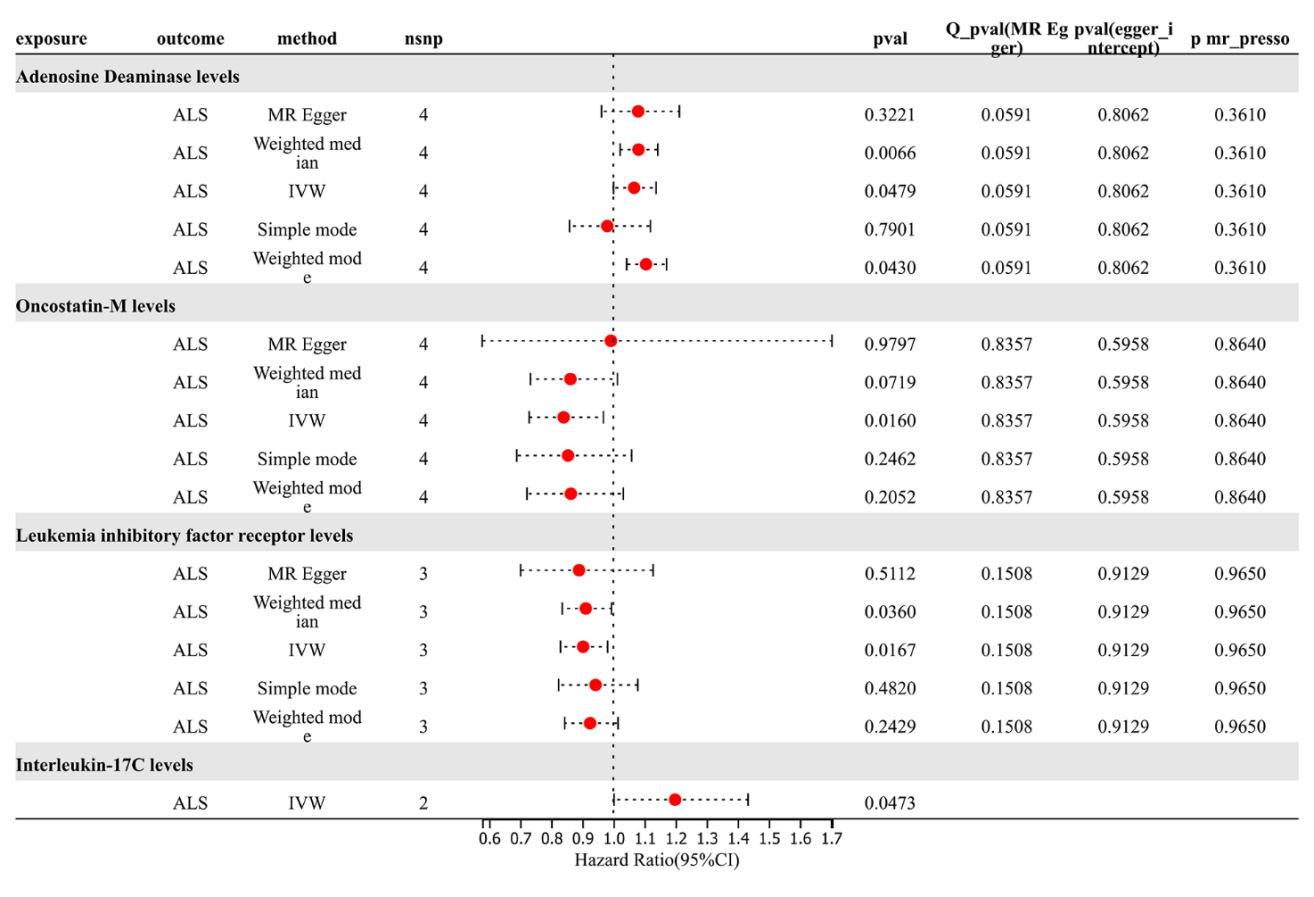
Forward MR results.

For analysis of TSMR results for inflammatory proteins and ALS, SNP (P < 5×10^-8) was chosen as the threshold to extract instrumental variables, and 73 inflammatory proteins were included in the TSMR analysis. The results indicated that elevated levels of Adenosine Deaminase increase the risk of ALS (OR=1.066, PIVW = 0.048). Elevated levels of Interleukin-17C were associated with an increased risk of ALS (OR=1.198, PIVW = 0.047) (SNPs < 3). Increased levels of Oncostatin-M were associated with a decreased risk of ALS (OR=0.840, PIVW = 0.016). Increased levels of Leukemia Inhibitory Factor Receptor were associated with a decreased risk of ALS (OR=0.903, PIVW = 0.017). The analysis indicated no significant heterogeneity (Q p-value > 0.05) or horizontal pleiotropy (P Egger Intercept > 0.05) for all the results (Figure 6 and Supplementary Table 7).

Subsequently, we conducted a leave-one-out analysis for the aforementioned 4 results, wherein each SNP was removed individually, and the effect size was estimated for the remaining SNPs. Notably, a deviation was observed in the results of Adenosine Deaminase levels upon the exclusion of SNP “rs112665079”, suggesting a significant impact of rs112665079 on MR estimation results. Furthermore, after the exclusion of rs112665079, Adenosine Deaminase levels showed no significant correlation with ALS in our MR analysis (Supplementary Table 8).

### Reverse Mendelian randomization results

**Figure 7:**
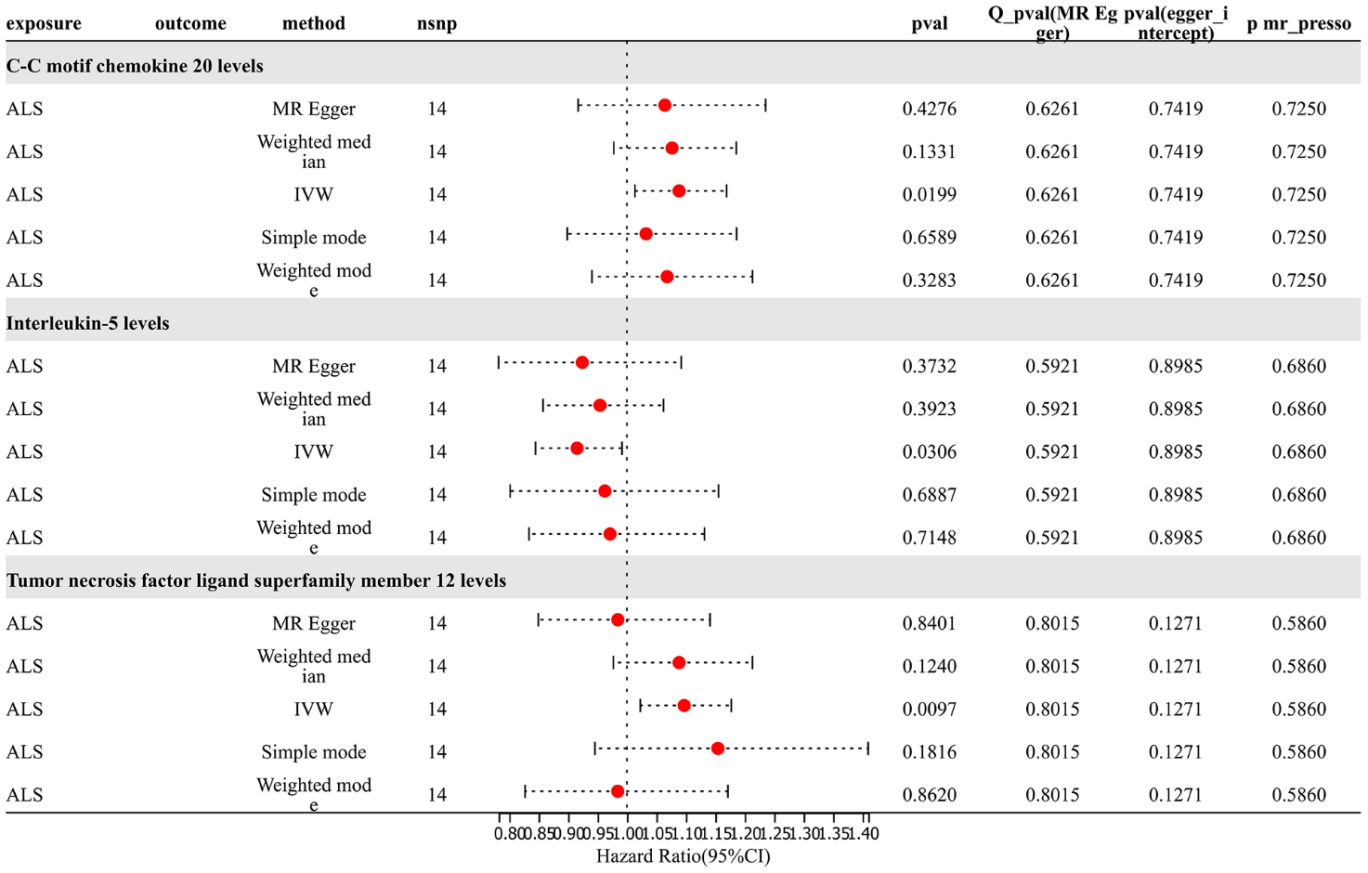
Reverse MR results.

For analysis of Mendelian randomization results for ALS and 91 inflammatory proteins, SNP (P < 5×10^-8) was chosen as the threshold to extract instrumental variables for TSMR analysis. The results revealed that an increased risk of ALS was associated with elevated levels of C-C motif chemokine 20 (OR=1.088, PIVW = 0.020). The risk of ALS increased with elevated levels of Tumor Necrosis Factor Ligand Superfamily Member 12 (OR=1.097, PIVW =0.010). The analysis indicated no significant heterogeneity (Q p-value > 0.05) or horizontal pleiotropy (P Egger Intercept > 0.05) for all the results. Conversely, an increased risk of ALS was associated with decreased levels of Interleukin-5 (OR=0.915, PIVW =0.031). Sensitivity analysis through leave-one-out method demonstrated the robustness of the results (Figure 7 and Supplementary Table 9).

## DISCUSSION

This study systematically evaluated the genetic correlation, genetic overlap, and causal relationship between circulating inflammatory proteins and ALS, exploring the relationship between inflammatory proteins and ALS from multiple dimensions. Bayesian colocalization analysis further enhanced the understanding of the interaction between inflammatory proteins and ALS. This may also reveal new therapeutic targets. By identifying proteins directly related to disease development, we provide a scientific basis for the future development of targeted therapeutic strategies. The methodology and findings of this study contribute to the realization of precision medicine. Through in-depth exploration of the molecular mechanisms of the disease and identification of specific biomarkers, it becomes possible to provide more personalized prevention, diagnosis, and treatment plans for ALS patients.

Our results indicate that certain circulating inflammatory proteins, such as CXCL1, IL18, LIFR, and CCL28, exhibit significant genetic correlation and overlap with ALS, which may unveil the potential role of inflammation in ALS pathology. Specifically, we identified a potential causal relationship between LIFR levels and ALS risk through TSMR analysis.

Our analysis also revealed some shared genetic loci, which may be key in the interaction between inflammatory proteins and ALS. Furthermore, we identified a shared pleiotropic SNP (rs10967965) in all trait pairs with positive genetic correlation and overlap, with NearestGene being MOB3B, located at 9p21.2, indicating its broad pleiotropy between various circulating inflammatory proteins and ALS. This suggests that these proteins may play crucial roles in the development of ALS. Moreover, our colocalization analysis revealed a potential pleiotropic locus (5q14.3), indicating its significant role in the correlation between the two diseases.

The NearestGene of rs10967965, MOB3B (MOB kinase activator 3B), is a protein that plays a role in various biological processes, including cell cycle regulation and cell death. Although there is relatively limited research on the specific role of MOB3B in neurodegenerative diseases, considering its potential role in regulating cell survival signals, MOB3B may play a role in ALS. Specifically, the rs10967965 locus may affect the expression or function of the MOB3B gene, thereby influencing neuronal survival and exacerbating the pathological process of ALS. Chromosomal regions 9p21.2 and 9q34.2 have been identified as regions containing ALS risk loci, highlighting the potential role of these regions in the pathogenesis of the disease. Chromosomal region 9p21 has been identified as an important genetic risk region in various diseases, including cardiovascular diseases and certain types of cancer. In conclusion, rs10967965 and its neighboring gene MOB3B may play a crucial role in the pathology of ALS, particularly in the mediation of immune circulating proteins such as LIFR, providing new research directions and potential therapeutic targets.

Our Mendelian randomization analysis further supported these findings, suggesting that changes in the levels of certain inflammatory proteins may affect the risk of ALS. For instance, we identified that LIFR has potential significance in genetic correlation, genetic overlap, and genetic causality with ALS. These findings provide important clues for future research and may contribute to the development of new therapeutic strategies.

LIFR plays a significant role in the nervous system and is associated with various neuropsychiatric disorders. LIFR, part of the interleukin 6 cytokine receptor family, is renowned for its involvement in cell differentiation, survival, and regeneration. In the nervous system, LIFR is crucial for neuron survival, neurogenesis, and neural plasticity [37–40].

Alterations in LIFR levels and signaling pathways have been observed in neuropsychiatric disorders. For instance, cytokine network dysregulation, including networks involving LIFR, has been reported in diseases such as depression, schizophrenia, and bipolar disorder. Such dysregulation impacts neuroinflammation, synaptic plasticity, and neuronal communication, contributing to the pathophysiology of these disorders [41, 42].

Furthermore, LIFR is involved in the process of neurodevelopment. Abnormalities in LIFR signaling can lead to impaired neural development, a key factor in the etiology of various developmental neuropsychiatric disorders. For instance, alterations in cytokine levels, including those related to the LIFR pathway, have been associated with developmental abnormalities in conditions such as Autism Spectrum Disorders and Attention Deficit/Hyperactivity Disorder (ADHD) [43, 44].

In neurodegenerative diseases such as Alzheimer's disease and Parkinson's disease, the role of LIFR in neuron survival becomes particularly crucial. LIFR-mediated signaling can affect neuroprotective pathways, and its dysregulation may contribute to the progression of neurodegenerative diseases. This opens potential therapeutic avenues, as modulating LIFR signaling could offer neuroprotective effects [45, 46].

Additionally, LIFR is involved in the response to neural injury. It promotes neuronal regeneration and repair, suggesting its potential therapeutic role in conditions like spinal cord injury or stroke [47, 48].

However, there is currently a lack of research on the relevance of LIFR in ALS. In addressing this complex disease, we believe that regulating the levels of inflammatory proteins may be a promising research direction. Specifically, research on LIFR can not only contribute to a deeper understanding of its mechanism of action in ALS but also lay the foundation for the development of new therapeutic strategies.

In conclusion, LIFR levels and signaling have complex connections with various aspects of neuronal function and are associated with a wide range of neuropsychiatric and neurodegenerative diseases. Understanding these relationships offers potential for developing new therapeutic strategies targeting the LIFR pathway. Furthermore, there has been no direct research focusing on LIFR and ALS to date. Building on this, our future research will focus on exploring the molecular and cellular biology mechanisms between LIFR and ALS, as well as validating clinical case data.

However, our study has several limitations. Firstly, our analysis is based on data from European ancestry, so the results may not be applicable to other racial or ethnic groups. Secondly, despite employing various statistical methods to bolster our findings, further research is still needed to confirm these results. Specifically, we need to conduct functional studies at the cellular and molecular levels to elucidate how circulating immune proteins like LIFR affect ALS. Additionally, cohort studies involving diverse ethnic populations are crucial to validate these results. Finally, clinical trials targeting these proteins can provide conclusive evidence of their therapeutic potential for ALS patients.

In summary, our study provides new insights into the genetic correlation and causality between circulating inflammatory proteins and ALS. These findings could help in understanding the pathogenesis of ALS and offer new directions for future therapeutic strategies.

## CONCLUSIONS

This study demonstrates significant genetic correlations and overlaps between certain circulating inflammatory proteins and ALS, suggesting potential causal relationships. These findings reveal the potential role of inflammation in the pathology of ALS and provide new insights for future research and therapeutic strategies.

## Supplementary Material

Supplementary Table 1

Supplementary Table 2

Supplementary Table 3

Supplementary Table 4

Supplementary Table 5

Supplementary Table 6

Supplementary Table 7

Supplementary Table 8

Supplementary Table 9
